# Physical Activity, Sedentary Behaviour and Metabolic Control following Stroke: A Cross-Sectional and Longitudinal Study

**DOI:** 10.1371/journal.pone.0055263

**Published:** 2013-01-29

**Authors:** Sarah A. Moore, Kate Hallsworth, Thomas Plötz, Gary A. Ford, Lynn Rochester, Michael I. Trenell

**Affiliations:** 1 Institute for Ageing and Health, Newcastle University, Newcastle upon Tyne, United Kingdom; 2 Institute for Cellular Medicine, Newcastle University, Newcastle upon Tyne, United Kingdom; 3 School of Computing Science, Newcastle University, Newcastle upon Tyne, United Kingdom; University of Queensland, Australia

## Abstract

**Background:**

Physical activity and sedentary behaviour are key moderators of cardiovascular disease risk and metabolic control. Despite the importance of a physically active lifestyle, little is known about the effects of stroke on physical activity. We assessed physical activity and sedentary behaviour at three time points following stroke compared to a healthy control group.

**Methods:**

Physical activity and sedentary behaviour were objectively measured using a portable multi-sensor array in 31 stroke participants (73±9 years, National Institute of Health Stroke Scale 2±2, mobile 10 metres with/without aid) within seven days and at three and six months. Stroke data were compared with an age, sex and body mass index matched healthy control group (n = 31).

**Results:**

Within seven days of stroke, total energy expenditure and physical activity were significantly lower and sedentary time higher in the stroke group compared to controls (total energy expenditure 1840±354 vs. 2220±489 kcal, physical activity 28±32 vs. 79±46 min/day, steps 3111±2290 vs. 7996±2649, sedentary time 1383±42 vs. 1339±44 min/day, p<0.01). At three months physical activity levels had increased (64±58 min/day) but plateaued by six months (66±68 min/day).

**Conclusions:**

Physical activity levels are reduced immediately post-stroke and remain below recommended levels for health and wellbeing at the three and six month time points. Clinicians should explore methods to increase physical activity and reduce sedentary behaviour in both the acute and later stages following stroke.

## Introduction

A physically active lifestyle reduces the risk of all-cause mortality and chronic disease, including stroke [1]. In contrast, physical inactivity (not a lack of physical activity but a prevalence of sedentary behaviour such as sitting or lying) reduces life expectancy and increases the risk worldwide of cardiovascular and metabolic diseases by up to 10% [2]. Physical inactivity is a commonly cited risk factor for stroke, with sedentary individuals 25–30% more likely to have a stroke than their physically active peers [3]. Despite the importance of a physically active lifestyle, little is known about the effects of stroke on physical activity and sedentary behaviour after stroke and then during recovery.

To date, research focussing on physical activity following stroke has used visual observation or accelerometry to report movement, both of which have limitations [4]–[7]. Observational studies have mostly been limited to a ward setting and used subjective recording of physical activity which is vulnerable to observer bias [4], [5]. In the community accelerometers have been demonstrated to be an accurate and reliable measure of step count following stroke [7]. This method, however, does not encompass non-stepping physical activity which may also play a role in health promotion [8] and the estimation of total energy expenditure from accelerometer counts has been found to be inaccurate [9]. Recently a portable multi-sensor array has been validated as an accurate measure of energy expenditure following stroke [10]. The multi-sensor array can be used to objectively capture physical activity in terms of energy expenditure and step count and allows for an exploration of sedentary behaviour.

Despite strong links between sedentary time and metabolic control [11], the role of sedentary behaviour has not been explored post stroke. As metabolic control is closely associated with increased stroke risk [3] this is an area worthy of study. Measuring how physical activity and sedentary behaviour change over time following stroke and the relationship between these measures and metabolic control may help guide therapeutic management following stroke and reduce the risk of stroke recurrence and other chronic disease.

The aims of this study were to describe: 1) physical activity, energy expenditure and sedentary behaviour at one week, three months and six months following stroke, comparing to an age, sex and BMI matched control group; and 2) metabolic control after stroke and the relationship between metabolic control, physical activity and sedentary behaviour.

## Subjects and Methods

### Ethics

The study was approved by the County Durham and Tees Valley Research and Ethics Committee on the 22/01/2010; all participants gave written informed consent. All clinical investigations were conducted according to the principles expressed in the Declaration of Helsinki.

### Participants

Thirty one people with stroke took part in this cross-sectional, longitudinal observational study. Participants were recruited from three stroke units within the National Institute for Health North East Stroke Research Network, between February 2010 and August 2011. Eligibility criteria included: 1) within seven days of stroke; 2) stroke diagnosis confirmed by a stroke specialist through computer tomography or magnetic resonance imaging and clinical characteristics; 3) >50 years of age; 4) independently mobile over 10 metres with/without walking aid; 5) mild-moderate gait deficit defined as asymmetry of gait (reduced stance time and increased swing time in the affected limb), or gait speeds of <1.3 m/s. Exclusion criteria included: 1) severe deficits in communication (inability to follow two stage commands); 2) mini mental state examination score <24; 3) mobility problems prior to stroke; 4) complicating medical history such as a co-morbid neurological disorder.

Healthy controls were recruited from the North East of England by newspaper advertisement and were matched to the stroke participants by age, sex and body mass index. Controls were free of diabetes and had no existing medical problems limiting physical activity.

### Outcome measurement

#### Stroke participants

Outcome measures were taken at one week, three and six months.

#### Objective physical activity/sedentary behaviour measurement

A validated portable multi-sensor array [10] (Sensewear Pro_3_, Bodymedia Inc, PA, USA) was used to gather physiological data on movement (via a bi-axial accelerometer), heat flux, skin temperature, and galvanic skin response. Data were converted using algorithms into: total energy expenditure (kcal/day); daily energy expenditure relative to baseline metabolism (kcal/kg/hour); physical activity/sedentary time (time spent doing activities >3/<3 metabolic equivalents (METS)); and daily steps. Absolute number of breaks in sedentary time was calculated by power law analyses of the lengths of sedentary bouts fitted from the raw sedentary data, as described in more detail previously [12]. The monitor was worn for seven days and was only removed for bathing.

#### Self-report physical activity measurement

The International Physical Activity Questionnaire (IPAQ) [13] was completed after wearing the multi-sensor array. Overall self-report physical activity score was calculated in metabolic equivalent (MET)-minutes per week from the IPAQ, alongside average daily sitting time [14].

#### Glycaemic control/lipid profile

A fasting (>8 hours) whole blood sample (approximately 5 mls) was obtained within seven days of recruitment. Plasma glucose, insulin and lipid profile were measured. Impaired fasting glucose and diabetes were categorised according to World Health Organisation guidelines [15] and dyslipidaemia according to Joint British Society guidelines [16]. The Homeostasis Model Assessment of Insulin Sensitivity (HOMA) (fasting insulin (mU/l)*fasting glucose (mmol/l)/22.5) was used to calculate insulin sensitivity, >3.0 indicated insulin resistance based upon previous population based studies [17], [18].

#### Control Participants

Control participants completed a past medical history questionnaire, were fitted with a multi-sensor array for seven days and then completed the IPAQ.

### Statistical analysis

Data were inspected for outliers using standard Z-distribution cut-offs and Mahalanobis distance tests respectively. Normality of distribution was assessed using a Kolmogorov-Smirnov test. Independent sample t-tests were used to test differences between stroke and control data if conforming to the assumptions of normality, if not the equivalent non-parametric Mann-Whitney U test was performed. A repeated measures within group analysis of variance was used to assess change in measures over time. Bivariate correlation was used to explore associations between physical activity and metabolic outcomes. Statistical analysis was carried out using SPSS version 17.0 (SPSS Inc. Chicago, Illinois, USA). Statistical significance was indicated if p<0.01 to allow for multiple comparisons. Data are presented as means ±SD unless otherwise indicated.

## Results

### Participant characteristics

**Figure 1:**
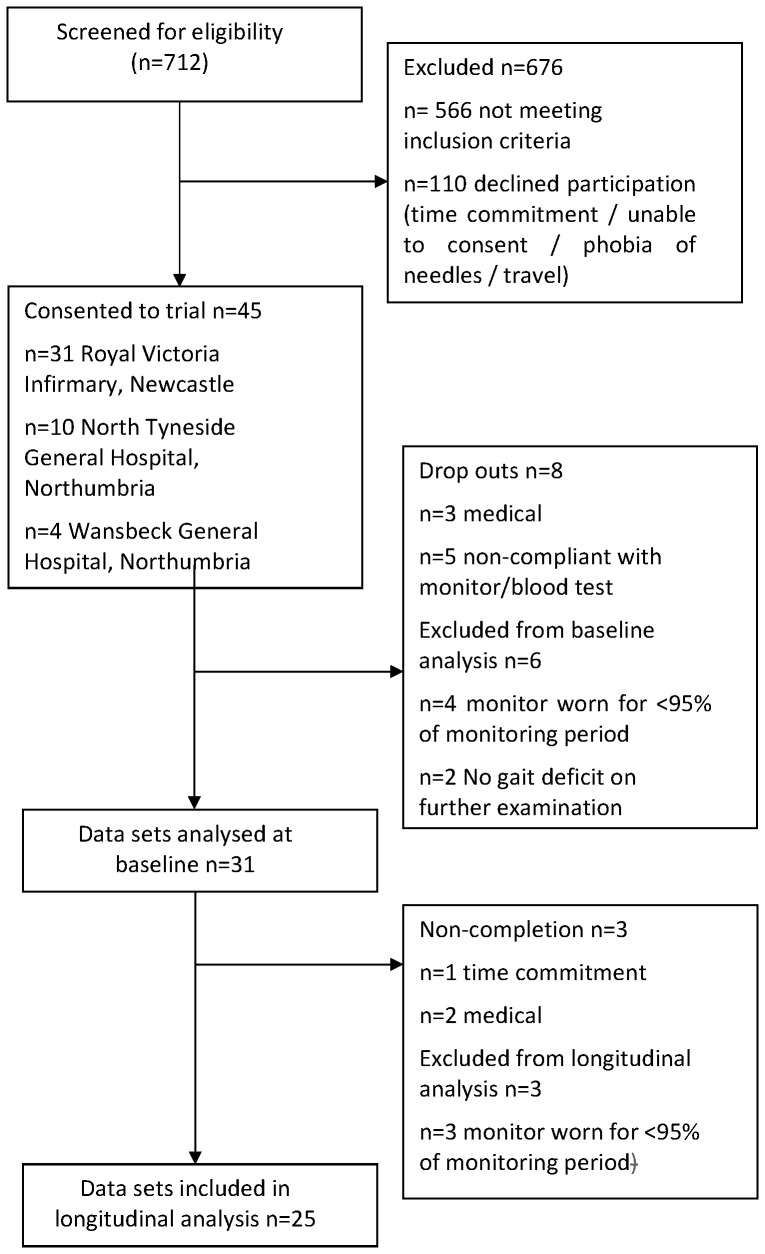
CONSORT flow diagram of trial recruitment, drop out and completion.

Following screening of 712 patients, 45 were consented into the trial with 31 participants completing baseline analyses and 25 completing all data time points (Figure 1 CONSORT flow diagram). Sex, age and body mass index were well matched between stroke participants and controls (Table 1). Stroke individuals had mild impairment (National Institute of Health Stroke Scale 2±2), slight disability (Modified Rankin Scale 2±1) and mild functional deficit (Barthel Index 93±10).

**Table 1:** Participant characteristics.

	Stroke (N = 31)	Controls (N = 31)	p value
Gender M/F	17/14	17/14	1.0
Age (years)	73±9	74±9	0.91
Height (cm)	167±9	166±11	0.92
Weight (kg)	76±16	70±12	0.10
Body mass index (kg/m^2^)	26.6±5.6	25.5±4.4	0.38
*Stroke characteristics*			
Stroke subtype (OCSP) N (%)			
Total anterior circulation	1 (3%)	-	
Partial anterior circulation	17 (55%)	-	
Lacunar	10 (32%)	-	
Posterior circulation	3 (10%)	-	
Infarct side L/R	15/16	-	
Stroke impairment			
NIHSS score	2±2	-	
Modified Rankin Scale	2±1	-	
*Functional characteristics*			
Barthel index	93±10	-	
Walking aid Y/N	2/29	-	
Walking speed m/s	0.8±0.3	-	

OCSP-Oxford Community Stroke Project classification, NIHSS- National Institute of Health Stroke Scale.

### Main results

Physical activity measures captured within seven days of stroke demonstrated participants (n = 31) were expending 19% (420 kcal) less energy per day in comparison to controls (n = 31), (1780±298 vs. 2200±489 kcal, p<0.01). Energy expenditure expressed as metabolic equivalents (METs) was 23% lower in patients than controls (1.0±0.2 vs. 1.3±0.1 kcal/kg/hr. p<0.01). Stroke participants only spent 27 minutes per day doing physical activity (>3 METS) and took 5040 less steps per day than their healthy counterparts (2956±2224 vs. 7996±2649 steps/day p<0.01). Stroke participants were sedentary (activity <3 METS) for 44 min/day longer than controls (1383±43 vs. 1339±44 min/day p<0.01).

**Figure 2:**
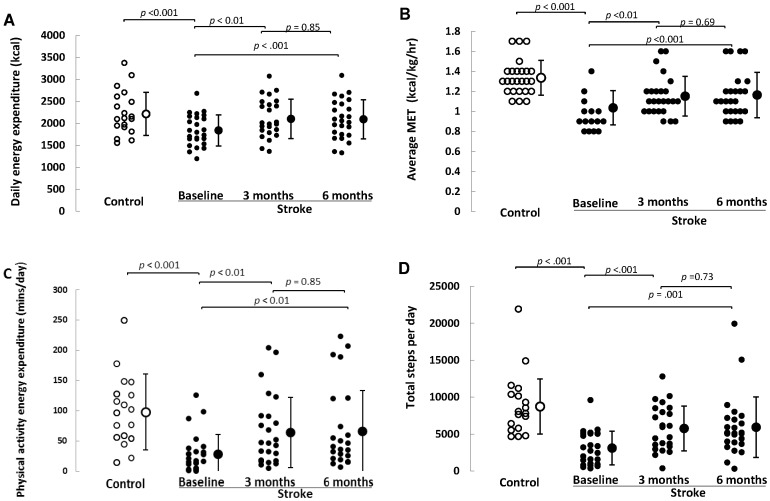
Physical activity levels following stroke with healthy control data for comparison. Energy expenditure (kcal/day) (Panel A); metabolic equivalent level (kcal/kg/hr.) (Panel B); physical activity duration (min/day) (Panel C) and daily step count (Panel D) in stroke participants within a week, at three months and six months following stroke in comparison to healthy control data.

The data from the 25 participants who were monitored at all three time points was then compared to 25 matched controls (Table 2 and Figure 2). The stroke subjects were less active on all physical activity measures taken within one week of stroke (p<0.01) spent more time sedentary (1383±43 vs. 1372±272 min/day, p<0.01) and had fewer breaks in sedentary time than the healthy controls (252±72 vs. 341±64, p<0.01). At three months stroke participants were taking fewer steps (5763±3026 vs. 8726±3735 steps/day, p<0.01), spending less time being physically active (64±58 vs. 98±63 min/day, p = 0.05), had lower average daily MET levels (1.15±0.2 vs. 1.3±0.2 kcal/kg/hr p<0.01) and had fewer breaks in sedentary time (291±65 vs. 341±64 p<0.05) than controls. At six months stroke participants still took fewer steps (5927±4091 vs. 8726±3735 steps/day, p = 0.02), had a lower average MET level (1.16±0.2 vs. 1.3±0.2 kcal/kg/hr., p<0.01) and had fewer breaks in sedentary time (282±62 vs. 341±64, p<0.01) than controls.

**Table 2:** Longitudinal physical activity, sedentary and metabolic measures after stroke with healthy control data for comparison.

Variable						Repeated measures ANOVA			
	Healthy control (n = 25)	Stroke 1 week (n = 25)	Stroke 3 months (n = 25)	Stroke 6 months (n = 25)	Main effect	Baseline-3 months		3–6 months	
						Δ	P value	Δ	P value
***Objective physical activity and sedentary measures (multi-sensor array)***									
Total energy expenditure (kcal)	2213±492	**1840±354** *	2100±447	2093±445	**<0.01**	**260**	**<0.01**	−7	0.85
Daily steps	8726±3735	**3111±2290** *	**5763±3026** *	**5927±4091** †	**<0.01**	**2652**	**<0.01**	164	0.73
Average MET (kcal/kg/hour)	1.3±0.2	**1.0±0.2** *	**1.15±0.2** *	**1.16±0.2** *	**<0.01**	**0.15**	**<0.01**	0.01	0.69
Physical energy expenditure (min/day)	98±63	**28±32** *	**64±58** *	**66±68**	**<0.01**	**36**	**<0.01**	2	0.85
Sedentary time (min/day)	1372±272	**1383±43** *	1350±57	1355±72	**<0.01**	**−33**	**<0.01**	5	0.64
Absolute number of breaks in sedentary time	341±64	**252±72** *	**291±65** †	**282±62** *	**<0.01**	**39**	**<0.01**	−9	0.11
***Subjective physical activity measures (International Physical Activity Questionnaire)***									
Energy Expenditure (MET-min/week)	4543±2742	**792±1155** *	**2608±3262** *	4665±13309	**<0.01**	**1816**	**<.001**	2057	0.46
Sitting (min/day)	289±129	**433±234** *	363±190	356±213	0.33	−70	0.27	−7	0.86
***Metabolic measure (fasting plasma samples)***									
Glucose (mmol/l)	-	5.5±1.2	5.2±0.7	5.3±0.6	0.23	−0.3	0.19	0.1	1.15
Insulin (mU/l)	-	11.3±12.5	9.9±6.2	9.3±6.3	0.44	−1.4	0.30	−0.6	0.34
Insulin sensitivity HOMA	-	2.6±5.5	2.6±1.6	1.9±1.6	0.70	0	0.73	−0.7	0.22
Cholesterol(mmol/l)	-	4.1±0.8	4.1±0.8	4.3±0.8	0.07	0	0.22	0.2	0.25
LDL-C (mmol/l)	-	2.2±0.7	2.1±0.7	2.2±0.8	0.67	−0.1	0.93	0.1	0.28
HDL-C (mmol/l)	-	1.3±0.2	1.4±0.3	1.5±0.3	**<0.01**	0.1	**0.02**	0.1	0.187

Significant differences between the healthy controls and the stroke participant data and longitudinal differences in the stroke data are indicated in bold:

* P<0.01;

† P<0.05. Metabolic measures above the normal values are underlined. Δ-Delta value, MET-metabolic equivalent.

In terms of longitudinal change following stroke, energy expenditure levels rose by 14% (260 kcal) between baseline and three months (p<0.001), but there was no further increase from three to six months (p = 0.85). Average MET level, physical activity duration (mins/day), daily step count and breaks in sedentary time all followed a similar pattern; increasing between baseline and three months (p<0.001), but plateauing between three and six months (Table 2).

### Metabolic control following stroke

Levels of total cholesterol and low density lipoprotein cholesterol were raised slightly at all-time points and insulin levels were raised at one week (Table 2). All other measures were within the normal limits. The only metabolic measure that rose over time was plasma HDL-C concentration (p<0.01).

A bivariate analysis determining if glycaemic and lipid control measures were associated with objective physical activity/sedentary measures revealed minimal relationships. The only significant relationships were at three months between glucose (mmol/l) (r = 0.55 p<0.01) and insulin sensitivity (HOMA) (r = 0.54, p<0.01) and total energy expenditure. There were no associations between change in physical activity and metabolic scores over the time points.

## Discussion

This is the first study to report objectively measured levels of physical activity, energy expenditure and sedentary behaviour over time following stroke compared to a healthy control group. The data reveals that within seven days of stroke physical activity is reduced and sedentary time increased in comparison to controls. Levels of physical activity increase three months post stroke without any further improvement to six months and remain below both the control group and recommended levels for health benefit. Longitudinal changes in physical activity were not related to metabolic control.

Within seven days of stroke individuals expend: 19% less total energy expenditure; are 66% less physically active; and take 63% fewer steps per day than matched controls. These objective data support observational studies reporting a high proportion of time on the ward is spent inactive following stroke [4]. Interestingly, the low level of physical activity cannot be solely attributed to impairment caused by the stroke as the group were all able to walk independently. The large variability in physical activity post-stroke between stroke units [5] also suggests that physical activity levels may not be fixed, but are determined by environmental factors ranging from access to therapists, to the physical design of the hospital environment. Importantly, those centres with lower levels of hospital based physical activity reported lower functional outcomes at discharge [19], although these were cross-sectional and not prospective studies. Combined these data suggest that, irrespective of its origins, low levels of physical activity are prevalent in stroke patients and may provide an important therapeutic target.

Physical activity levels significantly increased by three months, but plateaued by six months. Two previous stroke studies have prospectively monitored physical activity using the Step Activity Monitor. The first reported no significant change in steps per day over three time points (steps per day: 5541±1845 two days before discharge; 5506±2197 two weeks post discharge; and 6195±2195 six weeks post discharge p = 0.24) [6]. In contrast, the second study reported a significant increase in step count over time (1536±106 post discharge, 2765±1677 three months post discharge, p<0.001) [20]. The present study builds on these early observations. Caution, however, must be applied when comparing the findings of the different studies due to variability of placement of monitors and the fact the previous studies only collected data over a short period of time (<3 days) rather than over the recommended seven days [9] as in our study.

The plateau in physical activity at six months may suggest that patients have reached a stable pattern of everyday activity. Indeed, most patients are discharged from therapy services once independently mobile, with patients thought to reach a plateau in their potential at three months [21]. At this same time point, patients were only taking half of the 10,000 steps a day recommended for health and wellbeing [22]. It is not possible to determine if physical activity levels recorded were similar to those before stroke, or whether everyday activity was influenced by the stroke event itself. The low levels of physical activity observed post stroke, however, are particularly relevant because of the role of physical activity in both the primary [23], [24] and secondary prevention of cardiovascular disease [25] and stroke [26]. As a result, the low level of physical activity at six months post stroke, irrespective of whether or not it is reduced from pre stroke levels, will expose these patients to an excess risk of further cardiovascular complications.

In the present study, measures of metabolic control were not strongly related to physical activity levels and change in physical activity over time. This may have been due to our sample size being too small to show an association between metabolic control and physical activity and the fact metabolic measures were collected only in a fasting state and as such may lack the sensitivity required to track changes over time. Irrespective of the apparent lack of change in metabolic control, the data highlights that low levels of physical activity represent a sustained clinical burden post-discharge which is not being adequately addressed by clinical care.

To our knowledge this is the first study to objectively report sedentary behaviour following stroke. Immediately post stroke patients spent in excess of 23 hours per day sedentary, with this reducing fractionally to 22.5 hours of the day by three and six months. The physiological and behavioural implications of time spent sedentary are different to those of time spent being physically active. Time spent sedentary changes the hemodynamic stimuli that exert direct effects on the vasculature, leading to remodeling and a proatherogenic phenotype [27]. Longer bouts of sedentary time appear to have a negative impact on metabolic control, independent of the total amount of physical inactivity [28]. As a result, the reduced transitions from being sedentary to being physically active following stroke reported here may further exacerbate the impact of sedentary behaviour. As the positive health benefits of a physically active lifestyle appear insufficient to engage patients, it is possible that discussing the negative consequences of sedentary behaviour following stroke may provide an alternative perspective and facilitator on this important topic.

This is the first study to objectively capture physical activity and sedentary behaviour at three different time points following stroke, addressing some of the technical limitations of previous reports. The current data are, however, limited by the small sample size only representative of individuals with mild to moderate deficit. The monitor was placed on the non-hemiplegic arm as this has been found to be the most efficient place for capturing energy expenditure [29], therefore comparison of our results to lower limb mounted accelerometers or pedometers is problematic. Finally, the lack of pre-stroke physical activity data prevented assessment of the impact of stroke upon physical activity.

These data demonstrate that physical activity is significantly reduced post-stroke. Levels of physical activity increase by three months where they remain approximately 50% below national physical activity guidelines for health. The data also reveal that stroke patients spend around 23 hours a day sedentary. Understanding the deleterious effects of sedentary behaviour, as opposed to the benefits of physical activity, may provide new insights into stroke rehabilitation. Combined, these data highlight that clinicians and researchers should explore methods to increase physical activity and decrease sedentary behaviour in both the acute and later stages following stroke.
